# Partnering with Communities to Foster Trust, Save More Lives, and Prompt Recovery in Epidemics and Disasters

**DOI:** 10.1089/hs.2016.0092

**Published:** 2017-02-01

**Authors:** Monica Schoch-Spana

Beginning in 2017, new federal leadership can take important steps to vitalize the role of private citizens and businesses as well as faith-based and community-based organizations in the larger public health emergency preparedness (PHEP) enterprise. A broad consensus exists that government on its own cannot effectively and equitably manage epidemics and disasters. Past events repeatedly demonstrate that actions by citizens and civic groups have helped to curtail the impacts of extreme events and to prompt a more complete recovery from mass trauma. Nonetheless, the full potential of nongovernment forces in disaster readiness, response, and recovery has yet to be realized. Community engagement can enhance the quality of emergency planning, improve protections for vulnerable populations, multiply preparedness and response assets, and, ultimately, save more lives. The incoming administration in concert with Congress has an immense opportunity to enhance the country's resilience to catastrophic health events by steadily investing in robust partnerships between local public health authorities and the communities they serve.

## Recommendations

➢ **The Administration should use the bully pulpit to validate forcefully and consistently the whole community's role in helping manage disasters and epidemics.**

The new Administration should set the tone—to be carried on down to state and local elected officials and to their public health and safety advisors—that government should partner with private citizens, local communities, nonprofits, and businesses in the management of disasters and epidemics. Research suggests that the intangible quality of leadership plays a decisive role in helping build effective collaborations for population health. Strong, dedicated leaders who project a vision, inspire commitment, promote inclusion, empower staff, value innovation, and allocate resources have been an essential ingredient for successful private-public partnerships working on behalf of community health, including public health emergency preparedness.

Fueled in part by distorted news reports and Hollywood depictions, myths about unstable mobs in disasters linger, despite progress in thinking in recent years. Incoming leaders should preserve the gains made in understanding and capitalizing on citizen and civil society contributions during extreme events. Decision makers, for instance, should resist thinking that members of the public are panicking when they are merely engaging in understandable behaviors, such as searching for more information, questioning authorities, and adopting precautionary measures (even if officials judge these actions as unfounded). Research suggests that rather than being overwrought by an impulse for self-preservation at the expense of others, people engage in altruistic behaviors during disasters: Most people recovered from the wreckage of an earthquake, for instance, are saved by bystanders and nearby associates rather than search-and-rescue professionals.

Though an epidemic of novel disease may initially unnerve people, such reactions diminish as communities evolve coping strategies. Moreover, volunteers have played essential roles during major outbreaks of such scary diseases as smallpox, polio, Spanish flu, and HIV/AIDS—from staffing mass vaccination clinics, to helping the homebound, to influencing policies for disease prevention, vaccine development, and care delivery.

Federal leadership should model a resilience-building communication approach for decision makers at all levels to adopt throughout the crisis life cycle. Research suggests that during periods of everyday calm, people presume a state of safety and believe that a disaster will never happen, but if it did, it would affect others and not them. Leaders can temper this sense of invincibility with meaningful advice about proactive measures that people can take, individually and collectively, to diminish potential impacts: for example, family reunification plans, flood and earthquake insurance, neighborhood organizing, support for local bonds that underwrite structural mitigation projects.

During the crisis, top officials can avoid withholding information out of fear that people will panic, and instead provide steady updates, personal protective guidance, and details on volunteer opportunities to promote creative coping. Following the disaster, empathetic leaders can help ease distress by conferring meaning on the mass tragedy, focusing on the future, and underscoring collective learning. Moreover, they can take advantage of the window of opportunity that emerges when an otherwise theoretical threat has become a reality and people are primed to hear and act on prevention and mitigation advice.

➢ **The Administration should work with Congress to endow resource-starved local health departments with greater capacity to build community resilience partnerships**.

Government public health agencies are the glue that holds together society's preparedness and response system for disasters with health impacts. National thought leaders have consistently affirmed that the public health preparedness system includes individuals, communities, and businesses and that government public health agencies are accountable for system integration. Since the 9/11 and anthrax letter attacks, state and local public health departments have received PHEP financial and technical support from the federal government to prepare their jurisdictions to respond to disasters. Among the priorities of the PHEP grants program are risk communication; coalition building with business, faith-, and community-based organizations; incorporating public input into emergency plans and problem-solving sessions; and enlisting volunteers in everyday as well as emergency health and safety initiatives. Core principles of public heath practice further reinforce the obligation of state and local public health to work alongside and not independently of community members and their top health concerns.

To work *with*, not *for* the community in relation to preparedness and response, local health departments require sufficient organizational capacity. A key resource is a skilled, dedicated, and culturally competent staff who can develop an overall community engagement strategy, conduct broad public outreach and education efforts, design and deliver influential social media campaigns, and expend the face-time needed to build up trusted and lasting connections with grassroots groups and among vulnerable, underserved groups. A steady, ample budget is also necessary for successful community engagement. Expenditures include free training sessions and “giveaways” (eg, hand sanitizer, emergency backpacks) that hook people on preparedness; surveys, informant interviews, focus groups, translation services, and ethnic media “buys” that improve preparedness communication with diverse audiences; mini-grants to under-resourced organizations to catalyze interest in preparedness, raise employee and client preparedness levels, and support continuity of operations planning to meet client needs in an emergency; and formal processes to elicit public input into key policy decisions, such as how best to use scarce medical resources in a disaster. Agency leadership that prioritizes community engagement and inspires staff to work diligently on public involvement is another important ingredient for agency success.

Despite being a core feature of national health security, community engagement in emergency preparedness has been under-resourced, as part of a larger, perilous trend the new Administration should now turn around. In 2015, the National Association of County and City Health Officials (NACCHO) reported that local health departments had eliminated nearly 52,000 positions since 2008 due to layoffs and attrition in connection with hiring freezes or budget cuts. The Great Recession has produced lingering budget reductions, and nearly 1 in 4 local health departments is still affected by cuts today. In 2012, 15% of local health departments reported a reduction or elimination of emergency preparedness programs, on top of an earlier loss among 23% of local health departments in 2011. Federal PHEP funds have shown an overall trend of decline since the program's inception; in 2014, a majority of local health departments indicated their emergency preparedness funding comes only from federal sources. Community engagement is time and labor intensive work, yet it provides a high social return on investment. State and local health departments and their federal partners can benefit from having PHEP grant funding restored to original levels.

➢ **CDC should further motivate community- and faith-based organizations, especially those working among vulnerable populations, to integrate disasters into their mission.**

Vibrant grassroots organizations characterize every hometown: They give voice to collective needs, facilitate mutual aid, deliver social services, confer an identity and sense of belonging, and serve myriad other social functions and enjoyments. Neighborhood groups, senior centers, ethnic cultural centers, social service nonprofits, fraternal organizations, unions, faith communities, and other civic-minded associations can harness their leadership structures, communication networks, and command of local knowledge for the management of disasters and epidemics, thus complementing government's efforts. Among the roles that they can play are representing their members' interests in emergency planning sessions, amplifying and translating pre-event education and crisis communication messages, developing their own continuity of operations planning, providing emotional support amid mass tragedy, converting meeting halls into places of refuge, and rallying volunteers to backfill emergency response personnel during a crisis.

Past and present events underscore the important contributions of civil society groups in a crisis. During the 2014-15 Ebola outbreak, Translators Without Borders enlisted a network of volunteer translators to provide basic messages, posters, public service announcements, and “voice over” in the local languages for the most affected populations of Guinea, Mali, and Sierra Leone. After Hurricane Katrina in 2005, some New Orleans residents were wary of rebuilding because demolition, debris removal, and reconstruction seemed too daunting; exchanges among neighbors of labor, tools and equipment, expertise, shelter, and childcare, however, conveyed commitments to the community's future well-being and made rebuilding a material possibility. During the 1947 smallpox outbreak in New York City, officials vaccinated more than 6.3 million people over a 4-week period, enlisting private physicians, Red Cross volunteers, teachers' groups, women's clubs, and civil defense groups to staff free clinics in 12 hospitals, 84 police precincts, and every public and parochial school.

Similar to strengthening local health departments' ability to engage community partners, grassroots groups need sufficient capacity to work effectively with public health and emergency management agencies. The incoming Administration, via the PHEP grants program administered through CDC or even a novel program to strengthen the country's resilience from the bottom up, should provide financial and technical support to civic-minded groups for readiness, response, and recovery purposes. Should the proposed support be administered through the existing PHEP program in the form of mini-grants, health departments should receive adequate funding, grant-making flexibility, and a clear mandate to apportion capacity-building aid to their grassroots partners. Nonprofit groups may be unable to participate in meaningful disaster-related activities if it means diverting their otherwise scarce resources to yet another mission. Capacity-building resources should be targeted especially to nonprofits embedded in underserved minority populations, where cultural norms, language requirements, and social connections may not be well understood by mainstream institutions.

